# Vitamin-Containing Antioxidant Formulation Reduces Carcinogen-Induced DNA Damage through ATR/Chk1 Signaling in Bronchial Epithelial Cells In Vitro

**DOI:** 10.3390/biomedicines9111665

**Published:** 2021-11-11

**Authors:** J.P. Jose Merlin, Graham Dellaire, Kieran Murphy, H.P. Vasantha Rupasinghe

**Affiliations:** 1 Department of Plant, Food, and Environmental Sciences, Faculty of Agriculture, Dalhousie University, Truro, NS B2N 5E3, Canada; josemerlinj@dal.ca; 2 Department of Pathology, Faculty of Medicine, Dalhousie University, Halifax, NS B3H 1X5, Canada; dellaire@dal.ca; 3 Department of Medical Imaging, Faculty of Medicine, University of Toronto, Toronto, ON M5T 2S8, Canada; kieran.murphy@uhn.ca

**Keywords:** cancer, chemoprevention, gamma-H2AX, flavonoids, apple, quercetin

## Abstract

Lung cancer has the highest mortality rate worldwide and is often diagnosed at late stages, requiring genotoxic chemotherapy with significant side effects. Cancer prevention has become a major focus, including the use of dietary and supplemental antioxidants. Thus, we investigated the ability of an antioxidant formulation (AOX1) to reduce DNA damage in human bronchial epithelial cells (BEAS-2B) with and without the combination of apple peel flavonoid fraction (AF4), or its major constituent quercetin (Q), or Q-3-*O*-d-glucoside (Q3G) in vitro. To model smoke-related genotoxicity, we used cigarette-smoke hydrocarbon 4-[(acetoxymethyl)nitrosamino]-1-(3-pyridyl)-1-butanone (NNKOAc) as well as methotrexate (MTX) to induce DNA damage in BEAS-2B cells. DNA fragmentation, γ-H2AX immunofluorescence, and comet assays were used as indicators of DNA damage. Pre-exposure to AOX1 alone or in combination with AF4, Q, or Q3G before challenging with NNKOAc and MTX significantly reduced intracellular reactive oxygen species (ROS) levels and DNA damage in BEAS-2B cells. Although NNKOAc-induced DNA damage activated ATM-Rad3-related (ATR) and Chk1 kinase in BEAS-2B cells, pre-exposure of the cells with tested antioxidants prior to carcinogen challenge significantly reduced their activation and levels of γ-H2AX (*p* ≤ 0.05). Therefore, AOX1 alone or combined with flavonoids holds promise as a chemoprotectant by reducing ROS and DNA damage to attenuate activation of ATR kinase following carcinogen exposure.

## 1. Introduction

Lung cancer is the most diagnosed cancer worldwide and the leading cause of cancer-related deaths, largely due to late diagnosis and only partially effective chemotherapies [1,2]. Advanced treatments, such as surgery, chemotherapy, and radiotherapy interventions, have been shown to improve the survival rate of patients with primary lung cancer [3]. Chemotherapy is still the conventional treatment for lung cancer of the first and second stages. Advancements in immunotherapy have resulted in major improvements in clinical outcomes of lung cancer treatment [4]. For decades, there has been a link between cigarette smoking and the development of lung cancer. The risk of lung cancer is 6- to 10-times higher in smokers than in nonsmokers, and 90% of lung cancer patients are smokers [5,6]. According to the World Health Organization (WHO), chemoprevention is the most effective strategy for preventing lung cancer, particularly in smokers who already have pulmonary premalignancies [7]. 

4-[(Methyl)nitrosamino]-1-(3-pyridyl)-1-butanone (NNK) is a procarcinogen hydrocarbon found in cigarette smoke [8]. NNK is hydrolyzed by cytochrome P-450 enzymes into reactive metabolites, which covalently bind to DNA, forming DNA adducts and DNA double-strand breaks (DSBs) [9]. The phosphorylation of histone H2AX at serine 139 (γ-H2AX) and ataxia telangiectasia mutated kinase (ATM) at serine 1981 are induced by cigarette smoke, presumably as a result of NNK-induced DNA lesions [10]. Since NNK requires α-hydroxylation for metabolic activation, in cultured cell models, a precursor of NNK, 4-[(acetoxymethyl) nitrosamino]-1-(3-pyridyl)-1-butanone (NNKOAc), has been used to overcome the requirement of complex hepatic enzymatic activation of NNK [11]. Single-strand breaks (SSBs) are induced in a concentration-dependent manner by NNKOAc, which pyridyloxobutylates DNA and causes damage to all four bases [12]. Methotrexate (MTX) was discovered to induce genetic injuries such as chromosomal aberration, gene mutation, and DNA damage in various medicine toxicity tests [13,14]. MTX has also been linked to DNA damage in cancer cells, including oxidative damage in colon cancer and DSBs in non-small-cell lung cancer [15,16]. In our previous investigations, NNKOAc and MTX have been used to model smoke-related genotoxicity in normal human bronchial epithelial cells (BEAS-2B) [17].

Various intrinsic and extrinsic stresses, such as mutagenic chemicals, reactive oxygen species (ROS), ionizing radiation (IR), and unresolved intermediates of physiologic topoisomerase and nuclease reactions, cause DNA damage such as SSBs, DSBs, and base lesions, among others. The most dangerous DNA lesions that can cause oncogenic chromosomal aberrations are DSBs [18]. The PI3K-like kinases ataxia-telangiectasia mutated (ATM)-Chk2 and ATM-rad3-related (ATR)-Chk1 are the two major DNA damage response (DDR) transduction pathways for DNA damage repair [19]. ATR-Chk1 is activated by replication protein A (RPA)-coated SSBs, whereas ATM-Chk2 is activated by DSBs [20,21]. Following Chk1 and Chk2 activation, a variety of downstream effectors, including p53, γ-H2AX, BRCA1/2, and RAD51, are activated through post-translational modifications such as phosphorylation and ubiquitination. These processes have the potential to stop the cell cycle and trigger DDR mechanisms in the cell [22]. The apoptotic pathways are triggered when the insult exceeds the repair capacity to prevent the accumulation of DNA damage in cells that would otherwise lead to genomic instability and carcinogenesis. As such, activation of these DDR pathways represents a faithful read-out of genotoxic events experienced by the cell as well as its repair capacity. 

Due to their safety, low toxicity, and widespread availability, natural dietary antioxidants have the potential to suppress cancers and reduce the risk of cancer development by reducing cellular oxidative damage [23,24]. Antioxidants such as β-carotene, vitamin C, and flavonoids found in fruits and vegetables have anti-inflammatory, anti-diabetic, anti-fungal, anti-allergic, anti-viral, and anti-cancer properties [25]. Plant flavonoids and extracts protect cells from various forms of genotoxicity [26,27]. Quercetin has been shown to protect normal human bronchial epithelial cells (BEAS-2B) from Cr(VI)-induced carcinogenesis [28]. An apple peel flavonoid fraction (AF4) [17] and haskap berry flavonoids [8] were found to have antioxidant activity in an in vitro model to repair DNA damage in BEAS-2B cells. In the present study, we tested the hypothesis that a vitamin-containing antioxidant formulation (AOX1) that consists of vitamin C (ascorbic acid), vitamin B_9_ (folate), vitamin A precursor β-carotene, α-lipoic acid, and N-acetyl cysteine (NAC) alone or with the combination of AF4, quercetin (Q), or quercetin 3-*O*-d-glucoside (Q3G) could protect against DNA damage caused by selected chemical carcinogens, i.e., NNKOAc that mimic cigarette smoke-induced genotoxic damage in BEAS-2B cells. 

## 2. Material and Methods

### 2.1. Chemicals, Kits, and Antibodies

LHC-9 growth medium for BEAS-2B cells was purchased from Thermo Fischer (Chelmsford, MA, USA). The COMET SCGE assay kit for the comet assay was purchased from ENZO (New York, NY, USA). Cellular DNA fragmentation, the enzyme-linked immunosorbent assay (ELISA) kit for DNA fragmentation analysis, was received from Roche Diagnostics (Berlin, Germany). For γ-H2AX immunofluorescence studies, primary antibody anti-H2AX (S139) was obtained from Millipore (Etobicoke, ON, Canada), and secondary antibody Alexa Flour 594 donkey anti-mouse was purchased from Life Tech (Carlsbad, CA, USA). DNA Damage Antibody Sampler Kit (#9947) and β-actin (#12620) were purchased from Cell Signaling Technology (Boston, MA, USA). 4-[(Acetoxymethyl) nitrosamino]-1-(3-pyridyl)-1-butanone (NNKOAc) was purchased from Toronto Research Chemicals (Toronto, ON, Canada). Ascorbic acid, folic acid, β-carotene, α-lipoic acid, NAC, 3-(4,5-dimethylthiazol-2-yl)-5-(3-carboxy methyl phenyl)-2-(4-sulfophenyl)-2H-tetrazolium (MTS), phenazine methosulfate (PMS), dichlorofluorescin diacetate dye (DCFH-DA), and methotrexate (MTX) were purchased from Sigma-Aldrich (Oakville, ON, Canada). AF4 was isolated from peels of “Northern Spy” apples as described previously [29]. Stock solutions were prepared in dimethyl sulfoxide (DMSO), and the final concentrations did not exceed 0.5% (*v*/*v*) in the culture treatment medium.

### 2.2. Cell Culture 

BEAS-2B cells were purchased from the American Tissue Type Culture Collection (ATCC; CRL-9609) and were cultured with LHC-9 media at 37 °C in a humidified incubator with 5% CO_2_. Culture flasks (polystyrene T75) were pre-coated with a mixture of 0.01 mg/mL fibronectin, 0.03 mg/mL bovine collagen type I, and 0.01 mg/mL bovine serum albumin dissolved in LHC-9 medium overnight. Cells were cultured on the culture flask and grown to about 70% confluence, and the passages (<10) were used for all experimental conditions.

### 2.3. Cell Viability by MTS Assay

The cell viability assay (MTS) was used to determine the viability of BEAS-2B cells under different treatment conditions [8]. In order to determine the non-cytotoxic dose for the tested antioxidants, dose-dependent preliminary assays for various concentrations of the antioxidants were performed for 24 h [17]. Briefly, MTS reagent was added to each well and incubated for 3 h in the dark. Absorbance was recorded using a microplate reader (Infinite^®^ 200 PRO, TECAN, Mannedorf, Switzerland) at 490 nm. Cells with DMSO media were served as the vehicle control, and cells with medium containing MTS reagent were used as the blank for each experiment.

### 2.4. Measurement of Intracellular ROS

Intracellular ROS level in BEAS-2B cells was measured after adding DCFH-DA dye that was taken up by cells and hydrolyzed to DCFH, which can be oxidized by ROS to generate the fluorescent product dichlorofluorescein (DCF) [30]. The tested materials include the vitamin-containing antioxidant formula (AOX1) consisted of ascorbic acid, folate, NAC, lipoic acid, and β-carotene (10 µM each). For the comparison, AF4, Q, Q3G, and AOX1 with or without the combination of AF4, Q, or Q3G were used. Once the cells were pre-exposed to antioxidants for 3 h, they were then exposed to carcinogens for 3 h. Cells with only DMSO media served as the vehicle control (same treatment conditions were preserved for all experiments). After treatments, a final concentration of 5 μM DCFH-DA was added to the cells and incubated for 40 min in the dark. The fluorescence degradation was then measured at an excitation wavelength of 485 nm and an emission wavelength of 535 nm using a plate reader (Infinite 200 PRO, TECAN, Mannedorf, Switzerland). 

### 2.5. DNA Fragmentation Analysis

DNA fragmentation was measured by the cellular DNA fragmentation ELISA kit in BEAS-2B cells. Cells (1 × 10^5^ cells/mL) were labeled with 10 μM bromodeoxyuridine (BrdU), and 100 μL of BrdU-labeled cells were treated as mentioned above (Section 2.4). The cells were lysed with lysis buffer, and apoptotic DNA fragments in supernatants were collected for each sample after centrifugation at 250× *g* for 10 min. The samples (100 μL) were then transferred to 96-well flat-bottom microplates, which were precoated with anti-DNA and incubated for 90 min at 25 °C. The DNA was denatured by microwave irradiation (500 W for 5 min), then 100 μL of anti-BrdU-POD conjugate solution was added with an additional 90 min of incubation, and the plates were washed with wash buffer (1×) three times, and 100 μL of substrate 3,3′,5,5′-tetramethylbenzidine (TMB) solution was added for color development. The stop solution (25 μL) was added after 5 min, and the plates were read at 450 nm using a microplate reader (Infinite 200 PRO, TECAN, Mannedorf, Switzerland).

### 2.6. γ-H2AX Immunofluorescence Assay

The immunofluorescence method was used to measure the DNA damage at the histone level by quantifying γ-H2AX foci in BEAS-2B cells [31]. Briefly, 1 × 10^5^ cells were seeded on a coated coverslip placed in a 6-well plate with 24 h incubation. After treatments, cells were washed thoroughly with PBS and fixed with 3.7% formaldehyde and incubated for 20 min in the dark. The cells were then subjected to permeabilize with 0.5% Triton X-100 in PBS for 15 min at room temperature, which was followed by blocking with 4% BSA for 20 min. The cells were incubated with primary antibody (1:250) for 1 h at room temperature, washed three times with PBS, and then incubated with secondary antibody (1:500) for 45 min. The cells were washed with PBS three times, and the coverslips were carefully transferred onto the slides, and mounted by wet-mounting medium, Vectashield^®^-containing DAPI, and sealed with nail polish. The fluorescent images were captured by using a fluorescence microscope (EVOSTM FLoid Imaging System; Bothell, WA, USA).

### 2.7. Comet Assay

The comet assay, also called single-cell gel electrophoresis, was performed to measure the DNA damage [32]. Briefly, treated cells were combined with molten low melting agarose at a ratio of 1:10 (*v*/*v*), and 75 μL of each sample was pipetted to the slide and incubated for 20 min at 4 °C in the dark. The slides were then immersed in cold lysis buffer at 4 °C for 45 min and followed by alkaline solution for another 30 min in the dark. After washing the slides for 5 min with 1 × TBE buffer, they were subjected to electrophoresis (1 V/cm for 10 min). The slides were dipped in 70% ethanol for 5 min, allowed to air-dry, stained with CYGREEN^®^ dye (1:1000), and examined under fluorescence microscopy (EVOSTM FLoid Imaging System; Bothell, WA, USA). The comets were scored by using comet assay software (http://casplab.com/download, accessed on 14 July 2021), and a minimum of 30 cells were quantified by measuring the percentage DNA tail moment.

### 2.8. Western Blotting

BEAS-2B cells were harvested after the treatments and were lysed using 1 × SDS lysis buffer (1 mM Tris–HCl (pH 6.8), 2% *w*/*v* SDS, 10% glycerol) under reduced conditions on ice. The total protein concentration in each sample was measured by using the Bradford assay. A total of 20 μg of protein samples were loaded on 6% and 12% SDS-PAGE gel and electro-transferred to a PVDF membrane (Thermo Fischer). The membrane was then blocked with 5% non-fat milk solution for 1 h at room temperature and probed with specific primary antibodies (1:1000) for overnight incubation, washed and again probed with respective secondary antibodies (1:2000) for 1 h, and then developed by enhanced chemiluminescence (ECL) based on Clarity™ and Clarity Max™ (Bio-Rad, ChemiDoc^TM^ MP, Hercules, CA, USA). Protein expression of each band was normalized with the respective actin level, and relative protein expression was quantified with respect to untreated control bands for each experiment.

### 2.9. Statistical Analysis

All the experiments were performed in triplicates (*n* = 3) for three independent experiments and analyzed by one-way analysis of variance (ANOVA) using Tukey’s post hoc test and the two-tailed Student’s t test using GraphPad Prism 5 software (GraphPad software Inc., San Diego, CA, USA). Data were presented as mean ± standard deviation (SD) and *p* ≤ 0.05 was considered significant between experimental groups.

## 3. Results

### 3.1. Cell Viability Owing to Vitamins, AF4, Q, and Q3G Treatement

The effects of several vitamin/antioxidant preparations (AF4, Q, and Q3G) on cell viability were assessed in BEAS-2B cells by the MTS assay. The tested vitamins, flavonoids, and other antioxidants showed no cytotoxic effect at the concentration range of 0.1 to 1000 μM (Supplementary Figure S1A–E). At higher concentrations, Q (1000 μM), Q3G (250–1000 μM), and AF4 (above 12.5 μg/mL) were cytotoxic to BEAS-2B cells (Supplementary Figure S1F–H). Based on the % cell viability, 50 μg/mL of AF4 (cell viability above 80%), 50 μM of AOX1 (10 μM of each component), Q, and Q3G were selected for future experiments. All treatments were compared to a DMSO control with ≤5% cytotoxicity. 

### 3.2. Reduction of Intracellular ROS Levels by AOX1 Alone or with the Combination of AF4, Q, or Q3G

**Figure 1:**
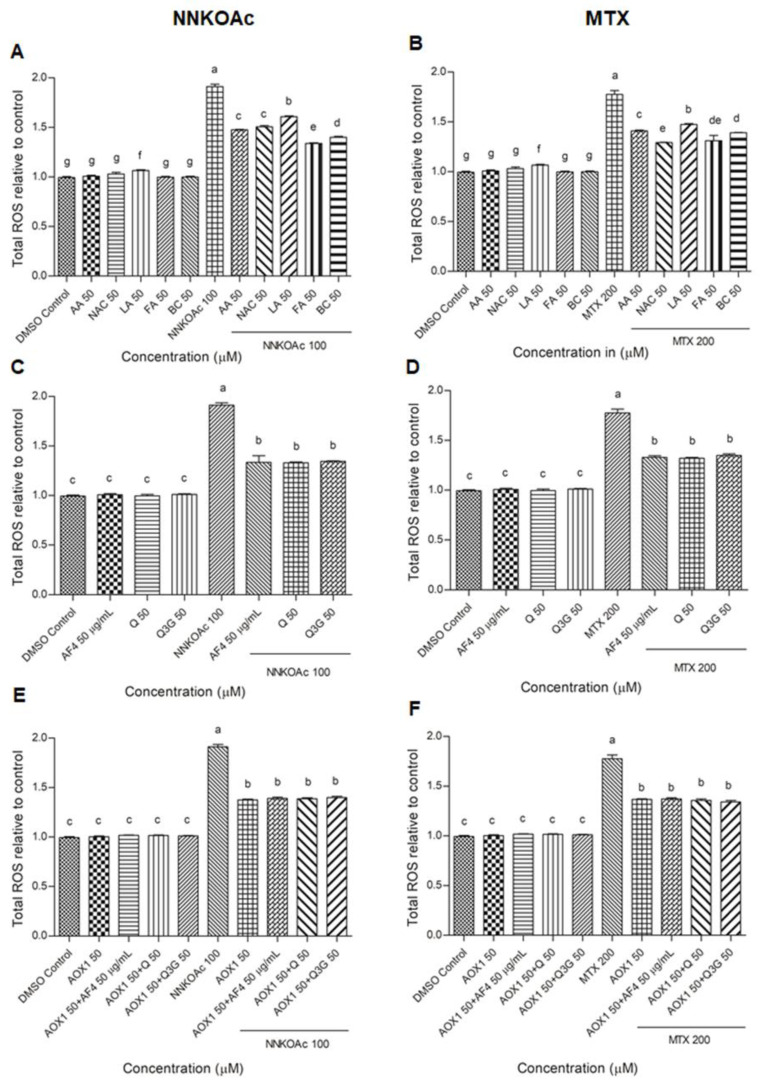
(**A**–**F**) Carcinogen-induced ROS production was reduced by pre-exposure of BEAS-2B cells with tested antioxidants. Experimental values presented as mean ± SD of *n* = 3 independent experiments by one-way analysis of variance performed with Tukey’s pairwise comparison. The sign “a,b,c,d,e,f, and g” refers to statistical difference (*p* ≤ 0.05). All the treatment groups were compared with the DMSO control. Means that share the same letter are not significantly different at *p* ≤ 0.05. Pre-exposure to vitamins, AF4, Q, Q3G, AOX1 with or without AF4, Q, or Q3G significantly (*p* ≤ 0.05) reduced ROS levels in BEAS-2B cells compared to NNKOAc-exposed cells. Abbreviations: AA: Ascorbic acid, AF4: apple peel flavonoid fraction 4, AOX1: antioxidant formulation, BC: β-carotene, FA: folic acid, LA: α-lipoic acid, MTX: methotrexate, NAC: N-acetyl cysteine, NNKOAC: 4-[(Acetoxymethyl)nitrosamino]-1-(3-pyridyl)-1-butanone, Q: quercetin, Q3G: Q-3-*O*-d-glucoside (Q3G).

In our previous study, we determined 200 μM of MTX and 100 μM of NNKOAc as the optimum concentrations for use in this cell model of carcinogen-induced DNA damage [17]. Using these compound concentrations, AOX1 and its five components, AF4, Q, Q3G, and their combinations were assessed using carcinogen-treated BEAS-2B cells to study the impact of dietary antioxidants on reducing intracellular ROS levels (Figure 1). The NNKOAc- and MTX-treated BEAS-2B cells showed a significantly greater (*p* ≤ 0.05) and a two-fold increase in total ROS level relative to the control. Pre-exposure to AOX1 components, AF4, Q, or Q3G significantly reduced (*p* ≤ 0.05) ROS levels in BEAS-2B cells compared to NNKOAc-exposed cells. Interestingly, all of the vitamins, AF4, Q, Q3G, and AOX1, as well as AOX1 with and without the combination of AF4, Q, or Q3G pre-treated cells, resulted in reduced levels of ROS compared to the carcinogen model in this study.

### 3.3. Protection from Carcinogen-Induced DNA Fragmentation in BEAS-2B Cells by AOX1 Alone and with the Combination of AF4, Q, or Q3G

**Figure 2:**
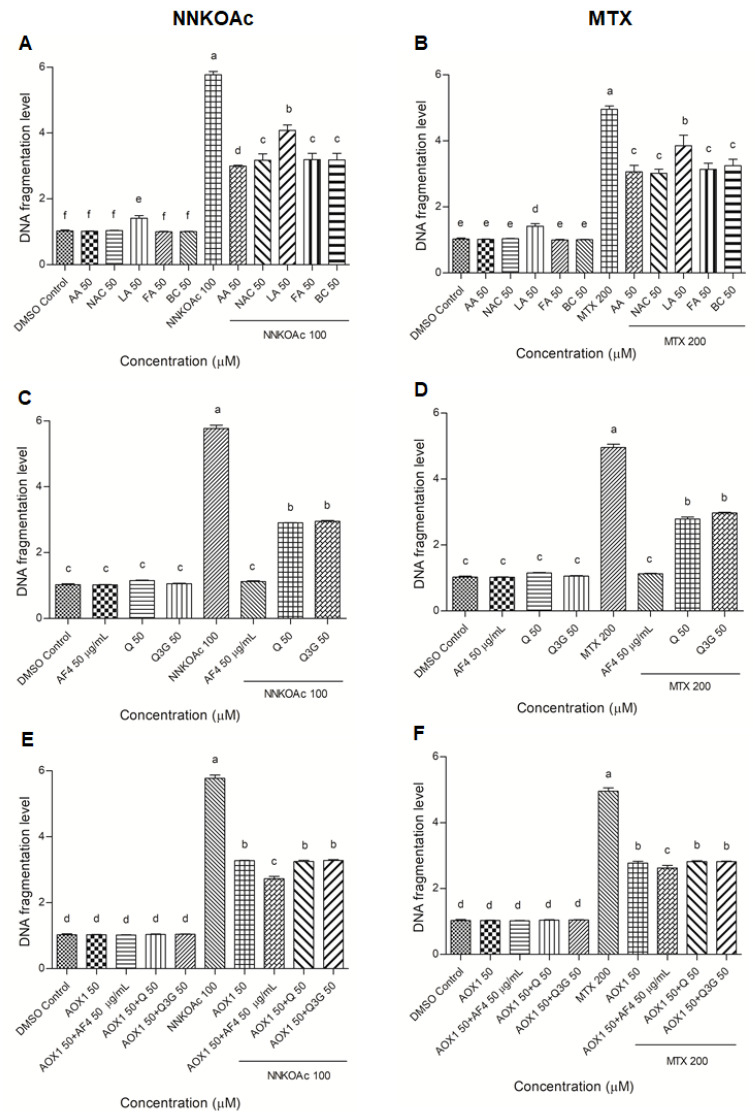
(**A**–**F**) DNA fragmentation caused by carcinogens in BEAS-2B can be reduced by pre-exposure of cells to tested antioxidants. Experimental values presented as mean ± SD of *n* = 3 independent experiments by one-way analysis of variance performed with Tukey’s pairwise comparison. The sign “a,b,c,d,e, and f” refers to statistical difference (*p* ≤ 0.05). All the treatment groups were compared with the DMSO control. Means that share the same letter are not significantly different at *p* ≤ 0.05. Pre-exposure to vitamins, AF4, Q, Q3G, AOX1 with or without AF4, Q, or Q3G significantly (*p* ≤ 0.05) reduced DNA fragmentation levels in BEAS-2B cells compared to NNKOAc-exposed cells. Abbreviations: AA: Ascorbic acid, AF4: apple peel flavonoid fraction 4, AOX1: antioxidant formulation, BC: β-carotene, FA: folic acid, LA: α-lipoic acid, MTX: methotrexate, NAC: N-acetyl cysteine, NNKOAC: 4-[(Acetoxymethyl)nitrosamino]-1-(3-pyridyl)-1-butanone, Q: quercetin, Q3G: Q-3-*O*-d-glucoside (Q3G).

The levels of DNA fragmentation in BEAS-2B cells were measured by the ELISA assay (Figure 2). When compared to the DMSO control, NNKOAc and MTX groups increased DNA fragmentation levels by five-fold (*p* ≤ 0.05). There was no DNA fragmentation caused by any of the AOX1 components (except lipoic acid), AF4, Q, Q3G, or AOX1. In carcinogen-treated groups, pre-treatment with AOX1 components, AF4, Q, Q3G, and AOX1, and AOX1 with and without the combination of AF4, Q, or Q3G, significantly (*p* ≤ 0.05) reduced DNA fragmentation.

### 3.4. Pre-Exposure to Antioxidants Reduces Carcinogen-Induced DNA Damage Indicator, Phospho-Histone Variant γ-H2AX

**Figure 3:**
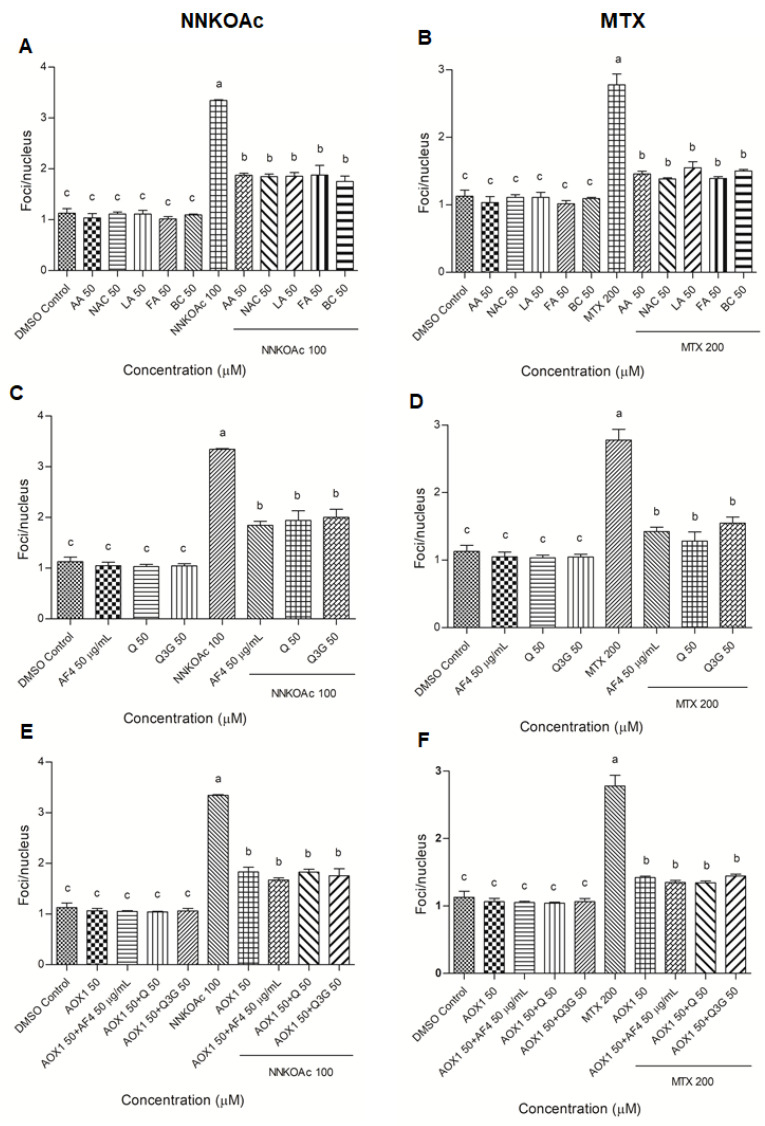
(**A**–**F**) Pretreatment of AOX1 alone or with the combination of AF4, Q, or Q3G reduced carcinogen-induced γ-H2AX in BEAS-2B cells. Quantification of focus/nucleus ratio was calculated for each sample from at least 30 cells. Experimental values presented as mean ± SD of *n* = 3 independent experiments by one-way analysis of variance performed with Tukey’s pairwise comparison. The sign “a,b, and c” refers to statistical difference (*p* ≤ 0.05). All the treatment groups were compared with the DMSO control. Means that share the same letter are not significantly different at *p* ≤ 0.05. Pre-exposure to vitamins, AF4, Q, Q3G, AOX1 with or without AF4, Q, or Q3G significantly (*p* ≤ 0.05) reduced DNA damage, as indicated by γ-H2AX levels in BEAS-2B cells compared to NNKOAc-exposed cells. Abbreviations: AA: Ascorbic acid, AF4: apple peel flavonoid fraction 4, AOX1: antioxidant formulation, BC: β-carotene, FA: folic acid, LA: α-lipoic acid, MTX: methotrexate, NAC: N-acetyl cysteine, NNKOAC: 4-[(Acetoxymethyl)nitrosamino]-1-(3-pyridyl)-1-butanone, Q: quercetin, Q3G: Q-3-*O*-d-glucoside (Q3G).

The results of the γ-H2AX immunofluorescence assay were used to examine DNA damage at the phosphorylation of histone protein on serine 139 (γ-H2AX; Supplementary Figure S2A–C). The nucleus (blue color) was stained with DAPI, which co-localized with γ-H2AX foci, which appeared red when observed under a fluorescence microscope. When compared to DMSO control cells, NNKOAc- and MTX-treated groups showed severe DNA damage (3-times higher). Treatment with AOX1 components, AF4, Q, Q3G, or AOX1 with and without the combination of AF4, Q, or Q3G did not cause any increase in DNA damage when compared to DMSO control cells. Pre-exposure to AOX1 individual components, AF4, Q, Q3G, or AOX1 alone or in combination with AF4, Q, or Q3G significantly (*p* ≤ 0.05) inhibited γ-H2AX foci/nucleus caused by NNKOAc and MTX exposure (Figure 3), consistent with reduced DNA damage (Figure 2).

### 3.5. Antioxidants Reduce Carcinogen-Induced DNA Damage Measured by Comet Assay in BEAS-2B Cells 

**Figure 4:**
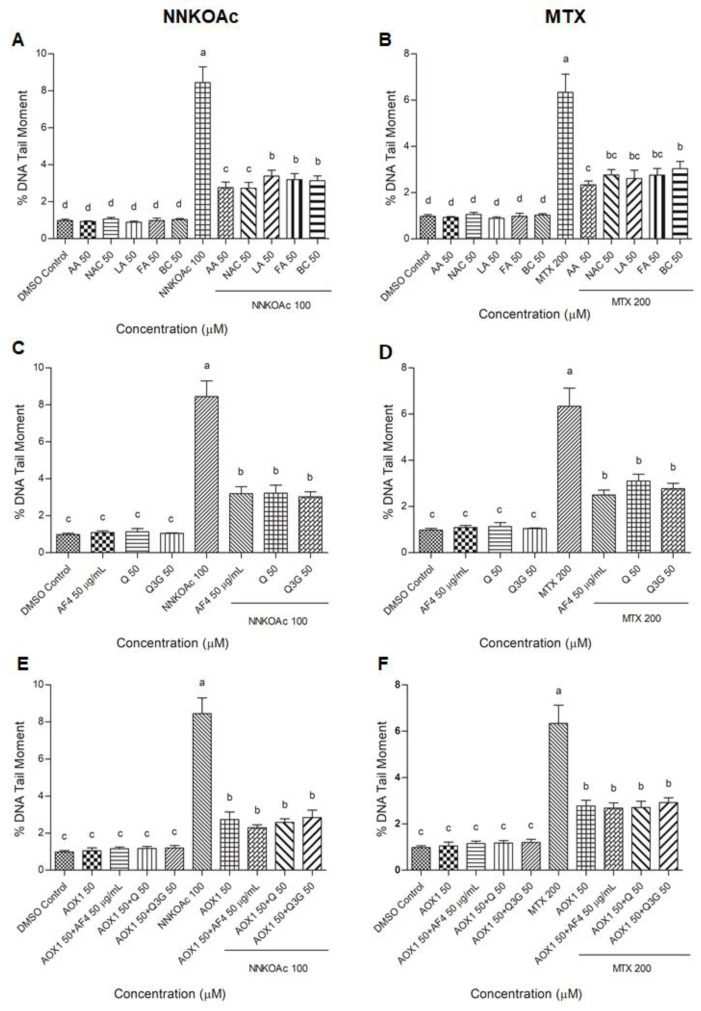
(**A**–**F**) Carcinogen-induced DNA tail damage measured by the comet assay in BEAS-2B cells is reduced by pretreatment of AOX1 with or without AF4, Q, or Q3G. Quantification of at least 30 comets were used for analysis and examined with a fluorescence microscope. Experimental values presented as mean ± SD of *n* = 3 independent experiments by one-way analysis of variance performed with Tukey’s pairwise comparison. The sign “a,b,c, and d” refers to statistical difference (*p* ≤ 0.05). All the treatment groups were compared with the DMSO control. Means that share the same letter are not significantly different at *p* ≤ 0.05. Pre-exposure to vitamins, AF4, Q, Q3G, AOX1 with or without AF4, Q, or Q3G significantly (*p* ≤ 0.05) reduced DNA damage in BEAS-2B cells compared to NNKOAc-exposed cells. Abbreviations: AA: Ascorbic acid, AF4: apple peel flavonoid fraction 4, AOX1: antioxidant formulation, BC: β-carotene, FA: folic acid, LA: α-lipoic acid, MTX: methotrexate, NAC: N-acetyl cysteine, NNKOAC: 4-[(Acetoxymethyl)nitrosamino]-1-(3-pyridyl)-1-butanone, Q: quercetin, Q3G: Q-3-*O*-d-glucoside (Q3G).

The comet assay is a well-established method of monitoring DNA damage and repair kinetics in cells by measuring the “tail moment” or the tail length × percentage of fragmented DNA migrating from the nucleus in the tail during in cellulo electrophoresis [33]. Following carcinogen treatments with and without vitamins/antioxidant preparations, DNA tail moment was measured (Supplementary Figure S3A–C and Figure 4). When compared to MTX treatment at the same concentration and exposure time, NNKOAc-treated cells showed a greater tail moment. Cells treated with AOX1 components, AF4, Q, Q3G, or AOX1 alone or with the combination of Q, Q3G, or AF4 maintained their cellular integrity and had a lower percentage of fragmented DNA in the tail. All the tested antioxidants and their tested combinations significantly (*p* ≤ 0.05) reduced the tail moment in BEAS-2B cells treated with either NNKOAc or MTX, as measured from at least 30 cells. 

### 3.6. Dietary Antioxidants Contribute to DDR Cell Signaling

**Figure 5:**
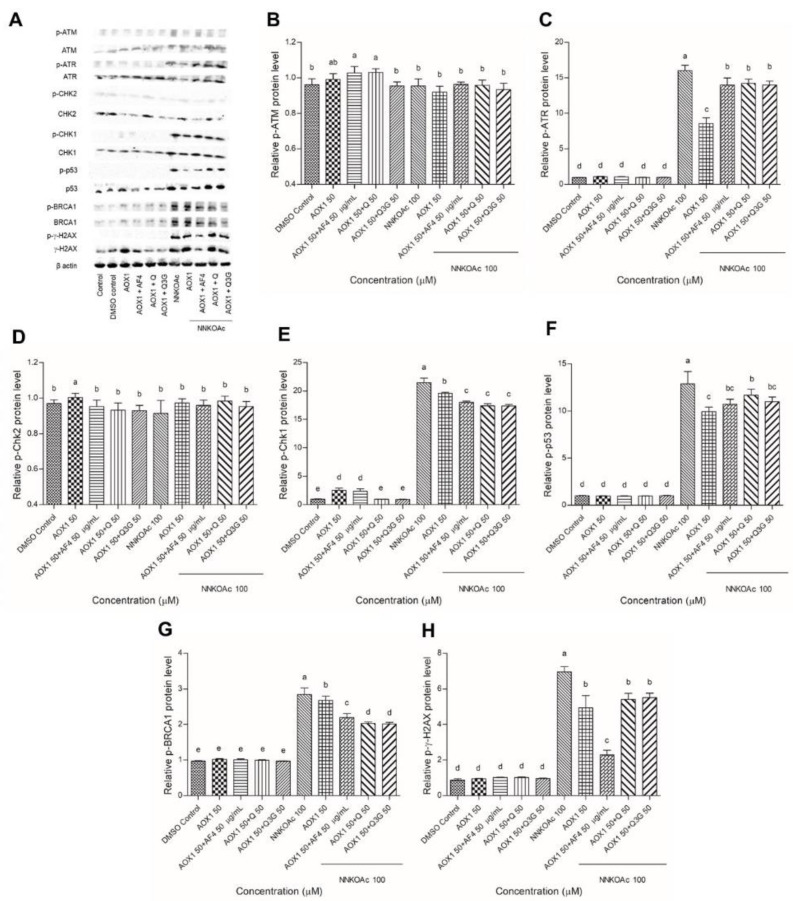
(**A**) Effect of AOX1 alone and with the combination of AF4, Q, or Q3G on various DDR signaling proteins exposed to NNKOAc was assessed by Western blotting. (**B**–**H**) The relative amount of each protein expression level (p-ATM, p-ATR, p-Chk2, p-Chk1, p-P53, p-BRCA1, and p-γ-H2AX) with respect to the beta-actin loading control. Experimental values presented as mean ± SD of *n* = 3 independent experiments by one-way analysis of variance performed with Tukey’s pairwise comparison. The sign “a,b,c,d, and e” refers to statistical difference (*p* ≤ 0.05). All the treatment groups were compared with the DMSO control. Means that share the same letter are not significantly different at *p* ≤ 0.05. Pre-exposure to AOX1 with or without AF4, Q, or Q3G significantly (*p* ≤ 0.05) reduced the protein expression levels of DDR in BEAS-2B cells compared to NNKOAc-exposed cells. Abbreviations: AF4: apple peel flavonoid fraction 4, AOX1: antioxidant formulation, NNKOAC: 4-[(Acetoxymethyl)nitrosamino]-1-(3-pyridyl)-1-butanone, Q: quercetin, Q3G: Q-3-*O*-d-glucoside (Q3G).

Western blot analysis was used to examine and quantify the phosphorylation of key upstream DDR kinases, ATM and ATR, and their effector cell cycle checkpoint kinases, Chk2 and Chk1, as well as tumor suppressor proteins p53 and BRCA1, and DNA damage molecular marker γ-H2AX (Figure 5). The DDR signaling cascade was enhanced in NNKOAc-treated cells, particularly ATR and its effector proteins, such as Chk1, p53, BRCA1, and γ-H2AX, which were all phosphorylated in response to these compounds. In NNKOAc-treated cells, however, phosphorylation of ATM or Chk2 protein was not observed. In NNKOAc-treated cells, pre-treatment with AOX1 with or without the combinations resulted in a significant (*p* ≤ 0.05) reduction in phosphorylation of ATR, Chk1, p53, BRCA1, and γ-H2AX. Overall, our findings revealed that the pre-treatment with AOX1 alone or in combination with Q, Q3G, or AF4 significantly reduced DDR protein phosphorylation and activation of the ATR/Chk1 axis, particularly in those challenged by NNKOAc-induced genotoxicity. 

## 4. Discussion

Many exogenous carcinogenic factors, including cigarette smoke hydrocarbons, cause DNA damage [34,35]. Normal cells are transformed into premalignant cells as a result of DNA damage and failure to repair, which result in mutations and abnormal cell proliferation [32]. In this study, we have used NNKOAc, a precursor of NNK that metabolizes into cytosolic reactive electrophilic metabolites, to induce DNA damage in BEAS-2B cells [36]. As an experimental model, we also employed MTX, a chemotherapy agent, to cause nuclear DNA damage in normal cells [17]. Cancer chemoprevention through dietary antioxidants has emerged as a promising medical intervention for lowering the risk of cancer development. Antioxidant-rich foods and dietary supplements can reduce oxidative nucleic acid damage [37]. In previous studies, we have demonstrated the selective cytotoxicity of AF4 to cause cell death in cancer cells with no or lower cytotoxicity to normal cells [38]. In BEAS-2B cells, we tested the cell viability of vitamins and antioxidant flavonoids at various concentrations and observed that there was no cytotoxicity at the doses employed that would be expected to be achieved in vivo after supplements. In humans, the plasma concentration of ascorbic acid ranges 40–80 μM [39], β-carotene is comparable to 2450 nM [40], folate is 0.9–55 nM [41], lipoic acid is 0.5–30 μM [42], and 141.5 μM for NAC [43]. However, we observed that Q and Q3G are toxic at higher concentrations. In contrast, we observed 80% cell viability for cells treated with 50 μg/mL AF4 treatment for 24 h, which is consistent with previous studies [17]. 

The exposure of BEAS-2B to NNKOAc and MTX resulted in the production of ROS and a reduction in cell viability. In normal healthy cells, ROS generation is one of the major contributing factors eliciting DNA damage [44]. Pre-treatment of BEAS-2B cells with tested antioxidants, AF4, Q, Q3G, and AOX1, and AOX1 combination with AF4, Q, or Q3G, significantly decreased the toxic effects of these carcinogens. Flavonoids are well-known for their ability to scavenge ROS [45]. In our previous study, the reduction of carcinogen-induced DNA damage by apple flavonoids (AF4) was shown [17]. AF4, Q, Q3G, and AOX1 components, as well as AOX1 with and without AF4, Q, or Q3G, have been found to protect BEAS-2B cells from carcinogens. 

In normal cells, DSBs represent some of the most difficult DNA lesions to repair. If DNA damage from DSBs is not repaired, it can lead to genomic instability and eventually carcinogenesis as DNA mutations accumulate [46]. Histone post-translational modification or γ-H2AX at the site of a DNA break is an early event in the DDR [47]. Phosphorylation of H2AX is essential in sensing and recruitment of DNA repair machinery to a wide range of DNA lesions [48]. After 3 h of exposure, the carcinogens used in this study were found to modulate H2AX phosphorylation status. Interstream crosslink-induced DNA damage, which is formed at replication forks and is largely responsible for observed γ-H2AX foci in NNKOAc-treated cells, could explain the observed toxicity [49]. Each DSB is assumed to be represented by a single focus [50]. Many studies have employed NNKOAc to overcome the limitations and complexity of NNK metabolism and to cause nuclear DNA damage in cells [8,12,17]. Carbonyl reduction, pyridine nitrogen molecule oxidation, and α-hydroxylation of the methyl or methylene carbons are the three main routes in NNK metabolism [51]. A considerable amount of NNK is converted to 4-(methylnitrosamino)-1-(3-pyridyl)-1-butanol (NNAL), a carcinogenic metabolite that is then oxidized to NNK via the carbonyl reduction pathway. CYP450 enzymes α-hydroxylate both NNK and NNAL, yielding electrophilic intermediates that can react with DNA to create bulky pyridyloxobutylation DNA (POB-DNA) adducts [52]. NNKOAc produces hydroxymethyl metabolites in cell culture systems, which spontaneously yield 4-3-pyridyl-4-oxobutane-1-diazohydroxide. POB-DNA adducts are formed when the diazohydroxides react with DNA. As a result, NNKOAc imitates NNK’s DNA damage pattern [12]. In addition to monitoring the formation of γ-H2AX foci, we also used the comet assay to examine DNA damage and fragmentation, which can detect both single-strand breaks and DSBs [53]. In this study, a quantitative analysis using the comet tail moment characteristics allowed us to monitor the severity of DNA damage caused by NNKOAc and MTX. Compared to carcinogen treatments, pre-exposure to tested vitamins, antioxidants, flavonoids, and AOX1 showed substantial inhibition of DNA tail damage. 

**Figure 6:**
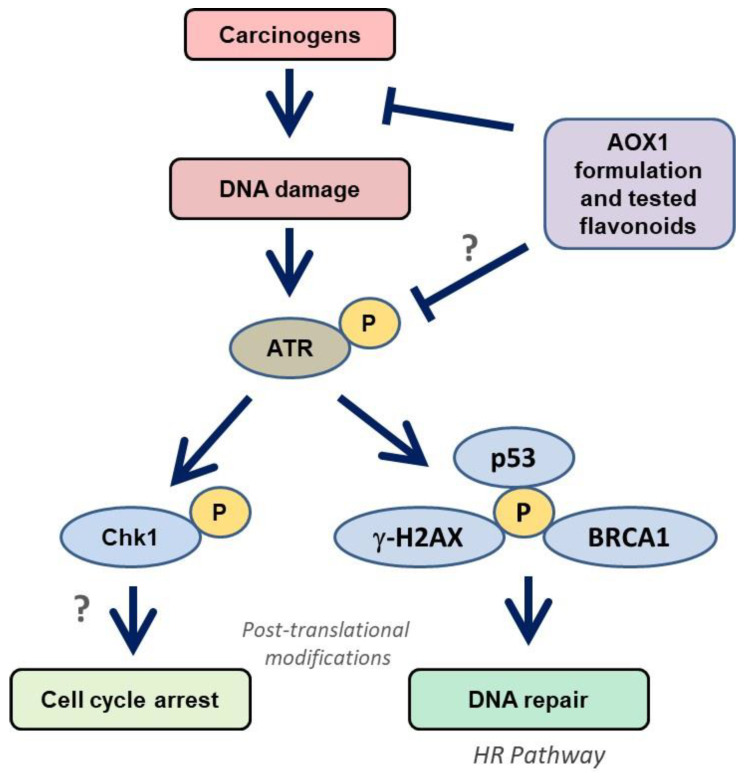
Modulation of ATR/Chk1 cell signaling pathway by AOX1 antioxidant formulation in BEAS-2B cells. ATR is activated by carcinogen-induced DNA damage and phosphorylates Chk1. The activation of ATR/Chk1 also initiates the DNA repair mechanism through p53/BRCA1/γ-H2AX signaling, which improves the HR repair mechanism. Abbreviations: AOX1: antioxidant formulation-1, ATM: Ataxia telangiectasia mutated, ATR: ATM-Rad3-related, Chk: checkpoint kinases, BRCA1: breast cancer gene 1, HR: homologous recombination, p53: tumor suppressor, ?: further studies are needed.

The recruitment of DDR factors to DNA damage was examined by immunoprobing against several proteins (ATM, ATR, Chk1, Chk2, p53, BRCA1, and γ-H2AX) in this study, which provides insights into the molecular mechanism of DNA damage generated by NNKOAc. DDR is made up of signaling pathways that coordinate replication, DNA repair, and cell cycle progression [54]. ATM-Chk2 and ATR-Chk1 are two essential signaling pathways in the DDR machinery. Mutations in the ATM-Chk2 pathway are common in malignancies, although mutations in the ATR and Chk1 genes are extremely rare [55]. This suggests that maintaining the activated ATR-Chk1 pathway is essential for cell survival [56]. Interestingly, our findings reveal that NNKOAc causes DNA damage, and this results in phosphorylation of ATR, but not phosphorylation of ATM in BEAS-2B cells (Figure 6). NNKOAc treatment also activated effector proteins such as Chk1, p53, BRCA1, and γ-H2AX. However, in BEAS-2B cells, MTX did not activate these proteins (data not presented). According to our hypothesis, MTX might be generating early events in DNA damage but may not be enough to initiate a cascade of effector proteins.

In our study, we observed that NKKOAc carcinogen-induced DNA damage activates ATR, which phosphorylates γ-H2AX and activates Chk1, initiating the DDR through various effector proteins such as p53 and BRCA1 to promote DNA repair by single-strand break repair and homologous recombination (HR) (Figure 6). In addition to replication stress, ATR is activated in response to UV-induced breaks, antimetabolites, inter-strand DNA cross-linking, and DSBs’ end resection [57]. Unlike ATM, ATR activation is usually triggered by single-stranded DNA (ssDNA). Furthermore, during the S or G_2_ phases of the cell cycle, DSBs are converted to ssDNA, which activates ATR [58]. Dietary antioxidants have been demonstrated to have chemopreventive effects via modulating a variety of signaling pathways, including the p53 pathway. In response to oxidative stress, p53 signaling activates transcription factors [59]. Dietary antioxidants can help to maintain oxidative homeostasis by phosphorylating and acetylating p53 protein. p53 regulates transcriptional genes involved in DNA repair, metabolism, senescence, apoptosis, autophagy, and angiogenesis via binding to DNA [60]. Our data indicate that ATR/Chk1 activation and signaling in response to carcinogen exposure is attenuated in response to the tested antioxidant formulations and likely reflects reduced ROS and consequently ROS-induced DNA damage.

Together, our findings support the ability of tested vitamins, antioxidants, flavonoids, and AOX1 to protect DNA damage in BEAS-2B cells. Polyphenols including luteolin, quercetin, and rosmarinic acid have similar effects in neuronal cells in protecting DNA from oxidative stress [19,60]. Sesaminol, a furofuran lignan derived from sesame seeds, enhances catalase and SOD activity and protects BEAS-2B cells from DNA damage caused by cigarette smoke extract [61]. β-Carotene is more sensitive to cigarette smoke-induced oxidation than lipids; however, the preferential oxidation of β-carotene in BEAS-2B cells did not result in a pro-oxidant effect [62]. The beneficial effect of lipoic acid on the activation of cytoprotective antioxidant genes makes it a potential option for reducing paraquat-induced oxidative stress-related bronchial cell death [63]. When BEAS-2B cells were transfected with γ-glutamyl transferase (GGT) cDNA, considerably larger amounts of ascorbic acid were deposited in the presence of glutathione (GSH), suggesting that active GGT may help in maintaining the ascorbic acid status of bronchial epithelia [64].

## 5. Conclusions

In this study, natural product formulations containing selected vitamins and antioxidant formulation used alone, or combination with flavonoids (AF4, Q, or Q3G), were able to protect BEAS-2B lung epithelial cells from chemical carcinogens’ insult in vitro. These results are a step towards developing therapeutic natural health products or dietary supplements to reduce not only the cancer risk of exposure to environmental carcinogens but also the adverse side effects of various cytotoxic or genotoxic chemotherapeutics. Such “natural-source” therapeutics may also prove useful in minimizing cancer risks associated with exposure to occupational hazards (e.g., diesel exhaust, smoke particles, and fumes) and diagnostic ionizing radiation exposure from radiography (X-rays), computed tomography (CT) scans, and nuclear medicine scans.

## Data Availability

The data that support the findings of this study are available from the corresponding author upon reasonable request.

## Supplementary Materials

The following are available online at https://www.mdpi.com/article/10.3390/biomedicines9111665/s1, Figure S1: The cell viability of antioxidant components on BEAS-2B cells, Figure S2: Immunofluorescence staining with and γ-H2AX antibody in BEAS-2B cells were exposed to carcinogens alone or in combination with pretreatment od antioxidant components, Figure S3: DNA tail damage in BEAS-2B cells were exposed to carcinogens alone or in combination with pretreatment of antioxidant components assessed by comet assay.

## Abbreviations

AA	Ascorbic acid
AF4	Apple peel flavonoid fraction 4
AOX1	Antioxidant formulation
ATM	Ataxia telangiectasia mutated
ATR	ATM-Rad3-related
BC	beta-Carotene
BEAS-2B	Normal human bronchial epithelial cells
Chk	Checkpoint kinases
DDR	DNA damage response
DMSO	Dimethyl sulfoxide
DSBs	DNA double-strand breaks
FA	Folic acid
LA	alpha-Lipoic acid
MTX	Methotrexate
NAC	N-acetyl cysteine
NNK	4-[(methyl)nitrosamino]-1-(3-pyridyl)-1-butanone
NNKOAc	4-[(acetoxymethyl)nitrosamino]-1-(3-pyridyl)-1-butanone
Q	Quercetin
Q3G	Quercetin 3-*O*-d-glucoside
ROS	Reactive oxygen species
