# Supplementary material for: Vitamin-Containing Antioxidant Formulation Reduces Carcinogen-Induced DNA Damage through ATR/Chk1 Signaling in Bronchial Epithelial Cells In Vitro
Source: Biomedicines. 2021 Nov 11;9(11):1665. doi: 10.3390/biomedicines9111665 (PMC8615515; doi:10.3390/biomedicines9111665)
Supplement: Supplementary file 1 [file biomedicines-09-01665-s001.zip › biomedicines-1426851-supplementary.pdf]

biomedicines

MDPI

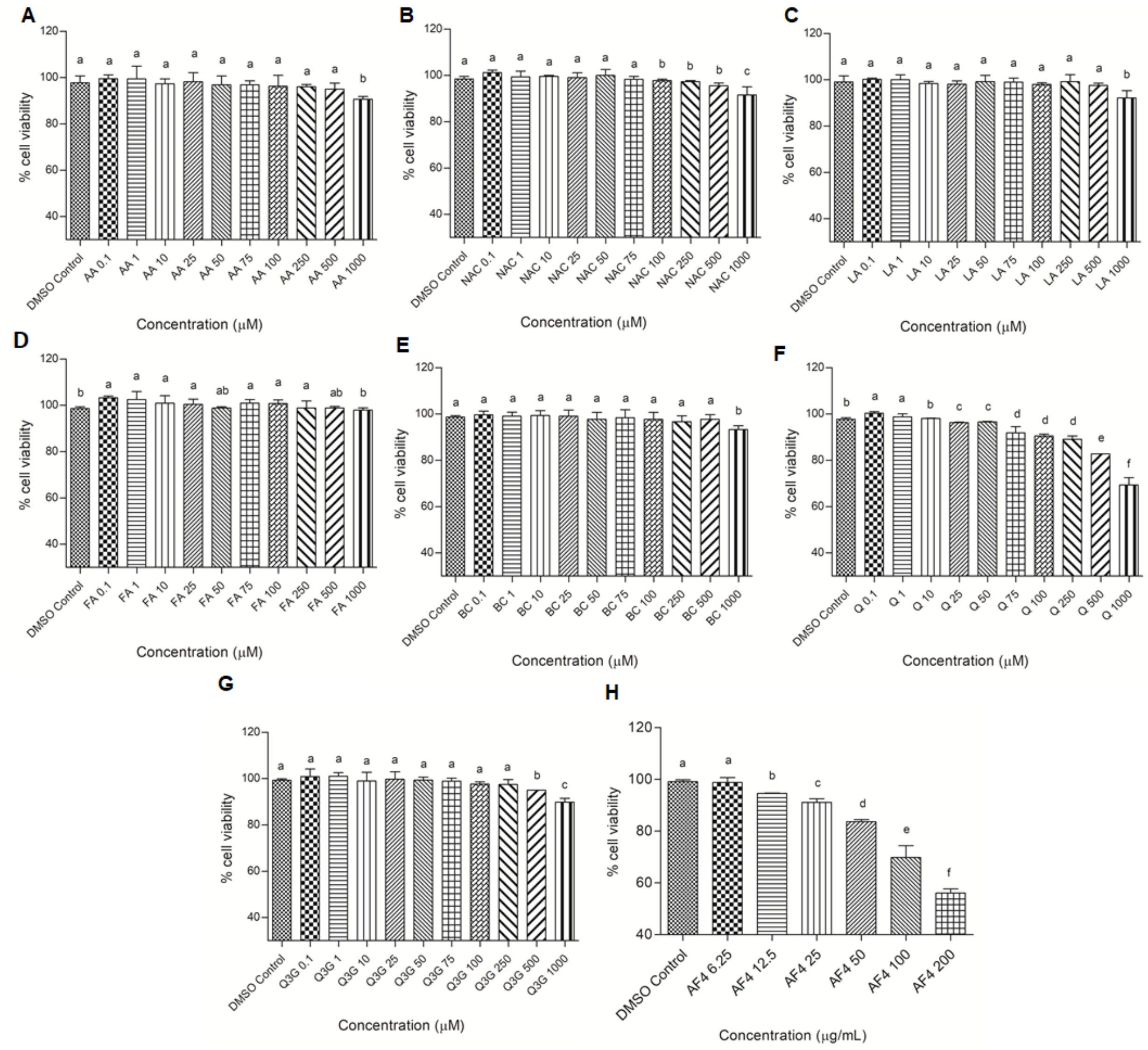

Figure S1. (**A**–**H**): The cell viability of Antioxidant components, Q, Q3G & AF4 on BEAS-2B cells after 24 h treatment. Experimental values presented as mean ± SD of $n = 3$ independent experiments by One Way Analysis of Variance was performed with Tukey's pairwise comparison. Means that share the same letter are not significantly different at $p \leq 0.05$. Abbreviation; AA: Ascorbic acid, AF4: apple peel flavonoid fraction 4, BC: β-carotene, FA: folic acid, LA: α-lipoic acid, NAC: N-acetyl cysteine, Q: quercetin, Q3G: Q-3-*O*-d-glucoside (Q3G).

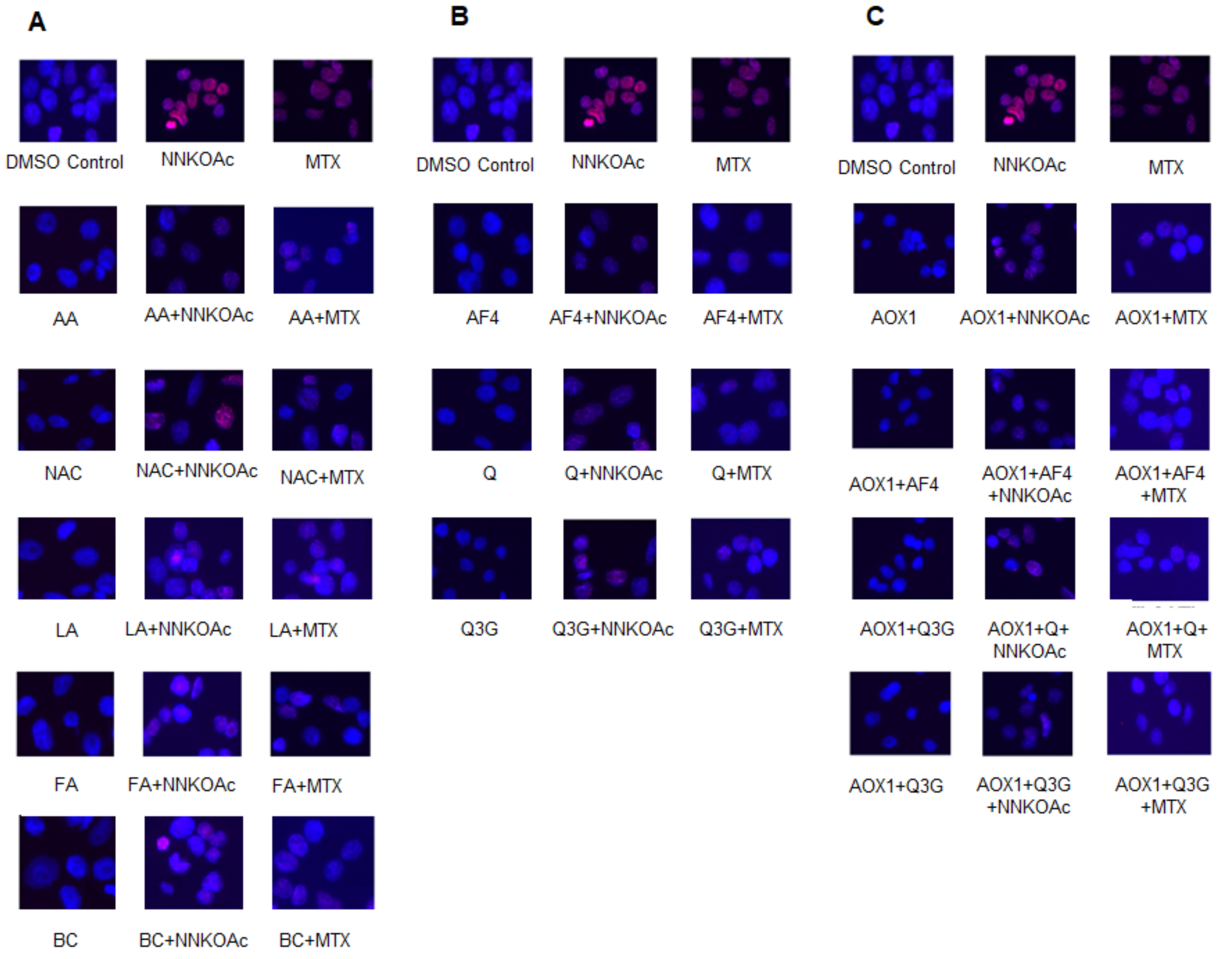

Figure S2. (**A**–**C**): BEAS-2B cells were exposed to either carcinogens alone or in combination with pretreatment of AOX1 alone and with combination of AF4, Q, and Q3G followed by immunofluorescence staining with γ-H2AX antibody and were captured by epifluorescence microscopy at 100x magnification. Nuclei were stained as blue and γ-H2AX foci (S 139) appeared as red. The image shown represents cells from three independent experiments. Abbreviation; AA: Ascorbic acid, AF4: apple peel flavonoid fraction 4, AOX1: antioxidant formulation, BC: β-carotene, FA: folic acid, LA: α-lipoic acid, MTX: methotrexate, NAC: N-acetyl cysteine, NNKOAC: 4-[(Acetoxymethyl)nitrosamino]-1-(3-pyridyl)-1-butanone, Q: quercetin, Q3G: Q-3-*O*-d-glucoside (Q3G).

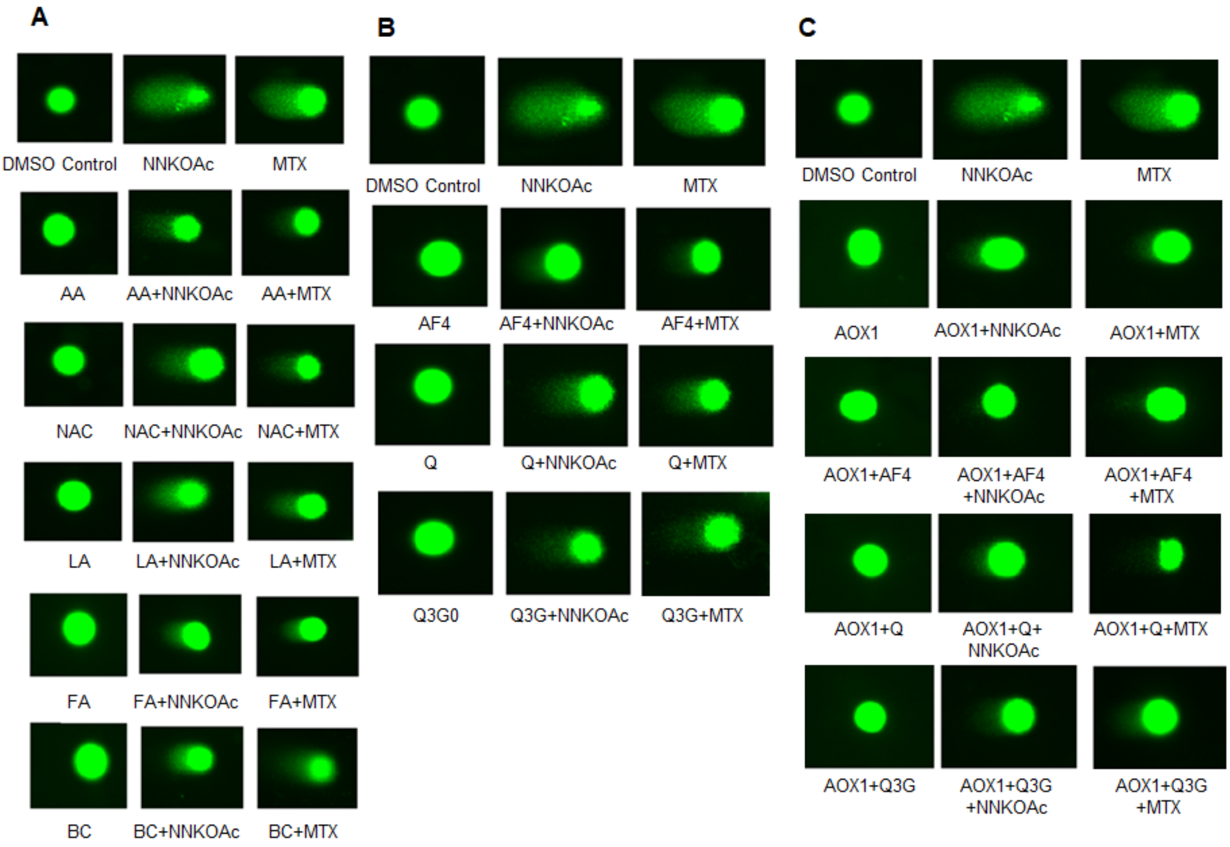

Figure S3. (**A**–**C**): DNA tail damage in BEAS-2B cells exposed to either carcinogen alone or in combination with pretreatment of AOX1 alone and with the combination of AF4, Q, Q3G, as assessed by comet assay. Abbreviation; AA: Ascorbic acid, AF4: apple peel flavonoid fraction 4, AOX1: antioxidant formulation, BC: β-carotene, FA: folic acid, LA: α-lipoic acid, MTX: methotrexate, NAC: N-acetyl cysteine, NNKOAC: 4-[(Acetoxymethyl)nitrosamino]-1-(3-pyridyl)-1-butanone, Q: quercetin, Q3G: Q-3-*O*-d-glucoside (Q3G).
